# Spatial analysis of visceral leishmaniasis in the oases of the plains of Kashi Prefecture, Xinjiang Uygur Autonomous Region, China

**DOI:** 10.1186/s13071-016-1430-8

**Published:** 2016-03-15

**Authors:** Li-ying Wang, Wei-ping Wu, Qing Fu, Ya-yi Guan, Shuai Han, Yan-lin Niu, Su-xiang Tong, Israyil Osman, Song Zhang, Kaisar Kaisar

**Affiliations:** National Institute of Parasitic Diseases, Chinese Center for Disease Control and Prevention, WHO Collaborating Center for Tropical Diseases; Key Laboratory of Parasite and Vector Biology, MOH, Shanghai, 200025 China; Center for Disease Control and Prevention of Xinjiang Uygur Autonomous Region, Urumqi, 830002 China; Center for Disease Control and Prevention of Kashi Prefecture, Kashi, 844000 China

**Keywords:** Xinjiang, China, Visceral leishmaniasis, Familial aggregation, Spatial aggregation

## Abstract

**Background:**

Kashi Prefecture of Xinjiang is one of the most seriously affected areas with anthroponotic visceral leishmaniasis in China. A better understanding of space distribution features in this area was needed to guide strategies to eliminate visceral leishmaniasis from highly endemic areas. We performed a spatial analysis using the data collected in Bosh Klum Township in Xinjiang China.

**Methods:**

Based on the report of endemic diseases between 1990 and 2005, three villages with a high number of visceral leishmaniasis cases in Bosh Klum Township were selected. We conducted a household survey to collect the baseline data of kala-azar patients using standard case definitions. The geographical information was recorded with GIS equipment. A binomial distribution fitting test, runs test, and Scan statistical analysis were used to assess the space distribution of the study area.

**Results:**

The result of the binomial distribution fitting test showed that the distribution of visceral leishmaniasis cases in local families was inconsistent (χ^2^ = 53.23, *P* < 0.01). The results of runs test showed that the distribution of leishmaniasis infected families along the channel was not random in the group of more than five infected families. The proportion of this kind of group in all infected families was 63.84 % (113 of 177). In the Scan statistical analysis, spatial aggregation was analyzed by poisson model, which found 3 spatial distribution areas 1) Zone A was located in a center point of 76.153447°E, 39.528477°N within its 1.11 mile radius, where the cumulative life-incidence of leishmaniasis was 1.95 times as high as that in surrounding areas (*P* < 0.05); 2) Zone B was located in a center point of 76.111968°E, 39.531895°N within its 0.54 mile radius, where the cumulative life-incidence of leishmaniasis was 1.82 times as high as that in surrounding areas (*P* < 0.01); and 3) Zone C was located in a center point of 76.195427°E, 39.563835°N within its 0.68 mile radius, where the cumulative life-incidence of leishmaniasis was 1.31 times as high as that in surrounding areas (*P* < 0.05).

**Conclusions:**

The spatial distribution of visceral leishmaniasis-infected families was clustered. Thus, the proper use of this finding would be an improvement in highly endemic areas, which could help identify the types of endemic areas and population at high risk and carry out appropriate measures to prevent and control VL in this area as well.

## Background

Visceral leishmaniasis (VL), also known as kala-azar, is a systemic disease caused by an intracellular protozoan belonging to the *Leishmania donovani* complex: *L. donovani*, *L.infantum* (which is also called *L.chagasi* and now regarded as synonyms), which are transmitted to humans by the bite of infected female phlebotomine sandflies [1]. The major features of VL are intermittent fever, enlargement of the spleen, and pancytopenia. The disease affects almost half a million people per year and is fatal if untreated [2–4]. A portion of apparently cured VL patients may develop post Kala-Azar Dermal Leishmaniosis (PKDL) as a sequel [5].

VL is still a serious public health problem in China and can be classified into two types based on the ecosystem and epidemiological characteristics [6–8]. The first type is a zoonotic one caused by *L.infantum* and it has been identified into two subtypes; namely a mountainous sub-type which is mainly distributed in Gansu and Sichuan Province and a desert sub-type endemic in the northwestern desert regions of China, including Xinjiang, western Inner Mongolia and northern Gansu. The second type is anthroponoticone which is endemic in the oases of the plains of Kashi Prefecture, Xinjiang Uygur Autonomous Region and is often prevalent in young people under 20 year-old. Some of the patients may develop post-kala-azar dermal leishmaniasis [9]. Concurrent cases of VL often occur in the same household in the endemic areas. The canine infection rate is usually very low (0–0.3 %) [10].

Kashi Prefecture has been one of the most seriously affected areas with anthroponotic VL in China. After a large-scale disease control carried out in endemic areas in 1950s, a remarkable achievement has been made. VL has been successfully controlled by 1958. Except for an individual case which was reported in the southern part of Hebei Province, there have been no new local cases reported in endemic areas since 1983. However, the number of cases and endemic foci for VL rose again starting in the 1990s. For instance, in 2003, a total of 321 cases were reported with a 92.87 % increase compared to 27 cases reported in 1990 [11].

A better understanding of the spatial distribution patterns of VL would provide information to help identify the types of endemic areas and population at high risk and carry out appropriate measures to prevent and control VL in this area. Such an idea motivated this study. The use of geographical information systems (GIS) with spatial statistics, including spatial cluster analysis has been applied to other diseases such as malaria, giardiasis, and schistosomiasis. This method is often used to analyze more clearly characteristic and the spatial patterns [12–14]. In this study, we aimed at performing a runs test, binomial test, and spatial Scan statistic with GIS to investigate geographical clusters of kala-azar in Kashi.

## Methods

### Ethics statement

Our project was initially approved by the Center for Disease Control and Prevention of Xinjiang Uygur Autonomous Region and Kashi Prefecture and local government. Then, this study was conducted according to the guidelines provided in the Declaration of Helsinki and all procedures were approved by the Ethics Review Committee of the National Institute of Parasitic Disease of China CDC. The written informed consents were obtained from all participants. For children under 10 years of age, informed consent had to signed by their legal representatives, and for children between 10 and 18 years, informed consent had to be co-signed by the children and their legal representatives.

### Study area and participants

**Fig. 1:**
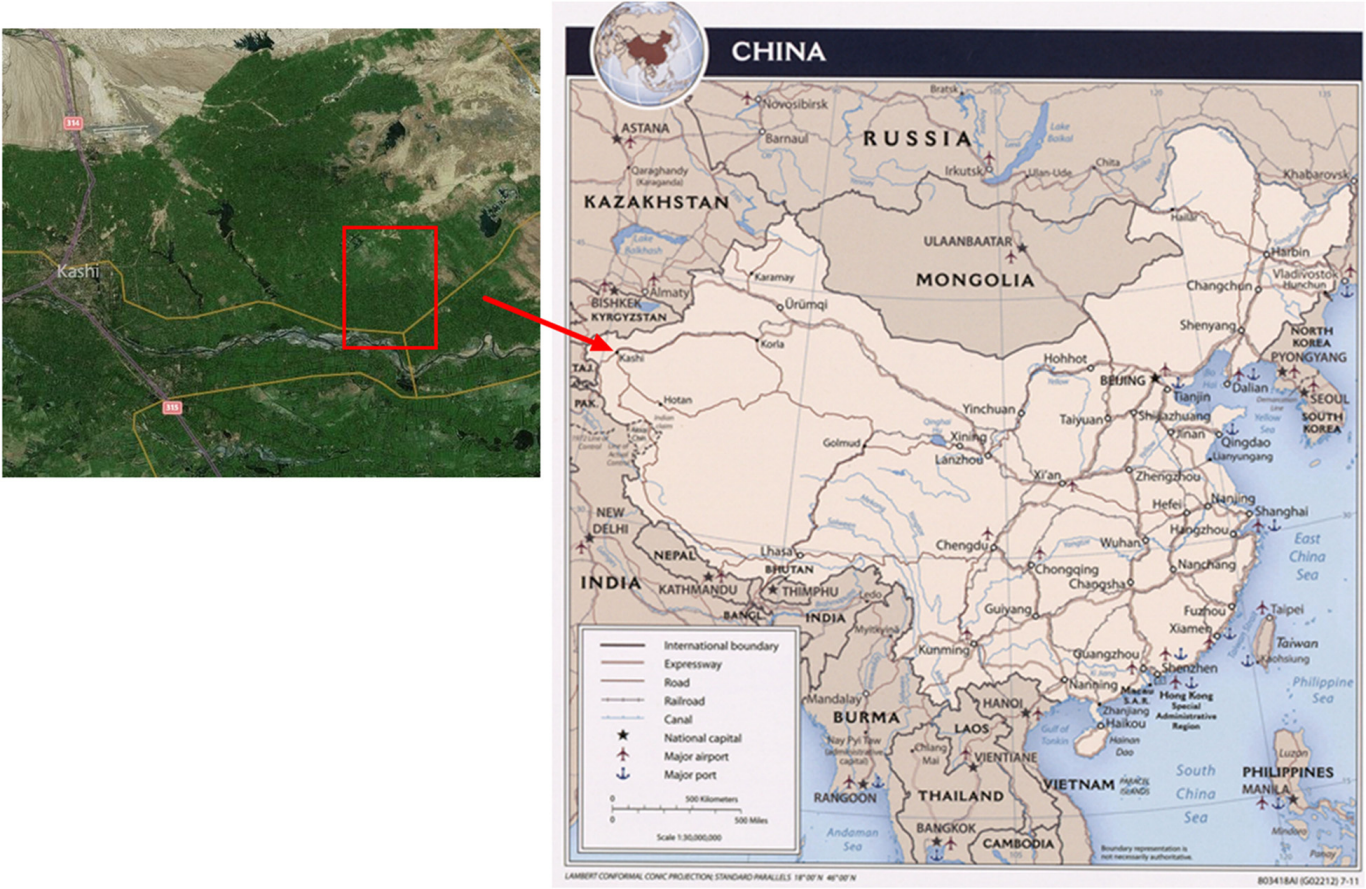
The map of study area. Red arrow and rectangle show the study area with respect to China

Bosh Klum Township was located in the east of Kashi Prefecture, which was in the south of Xinjiang Uygur Autonomous Region, as shown in Fig. 1. According to the report of epidemic diseases between 1990 and 2005, three villages with a high number of visceral leishmaniasis (VL) cases in Bosh Klum Township were selected; these were the 1st village, 18th village, and 20th village. Anthroponotic kala-azar was only endemic in Kizilsu Kirghiz Autonomous Prefecture, Kashi Prefecture, and Shufu Delta in the south of Xinjiang Uygur Autonomous Region [15]. Bosh Klum Township of Shufu County was the highest incidence area with kala-azar. Since the 1990s the number of cases in this county stayed at a high level, and the new cases of the three selected villages in 2003–2004 accounted for 43.53 % (74/170) of the total cases reported by 21 townships in the whole county.

The local residents in Bosh Klum Township served the brigade as the basic administrative units, where the geographic position of every resident is relatively concentrated. Generally, different brigades were separated depending on crops and/or fruits planted in a specific region leading to the divided and independent geographic positions between brigades. A total of 24 brigades in the 3 villages were at census points. All the children were enrolled in the project.

Other factors were used to group houses in the study area, for example, households along the irrigation canal were regrouped and resident houses which were along the same ditch were designated into the same group.

### Data collection

Data was obtained by 24 teams from a house-to-house survey. People enrolled in the study were interviewed by trained health professionals using standardized questionnaires that included demographic information (such as names of family members, age, occupation, educational level) and VL history. The questionnaires were pre-tested and translated into the local language. The location of each family from the selected three study villages was measured with hand-held GPS. Thus, the average geographic coordinate of all the family accommodated in each team were calculated and taken as the center coordinate of the specific team. The geographical information of roads in the investigation area was recorded by vehicle-mounted GPS mobile station and satellite, and their error was corrected by real-time records from GPS station.

The identification criteria of kala-azar patients used in this survey were as follows:1) if someone visited the designated medical institution which could make a diagnosis of kala-azar (e.g., District Center for Disease Control and Prevention) and was diagnosed with kala-azar; or 2) if someone received the treatment of Sodium Stibogluconate, which made symptoms improve. A respondent who was in accordance with the above two criteria were identified as kala-azar patient.

### Statistical analysis

Database was established using MicrosoftAccess™ 2003. The double entry and validation approaches were used to ensure the quality of data entry using Epi-info™ 3.4.3 (Centers for Disease Control and Prevention, USA). Statistical analysis was performed using SPSS™ 21.0 (IBM). The extraction maps were obtained and the geographic information database was established using ArcGIS™ 10.1 (Esri).

Goodness-of-fit test of the binomial distribution was used to make a preliminary understanding of the households cluster in this area. Furthermore, we used a run tests to measure the randomness of arrangement of VL families. Finally, since the annual incidence of VL was usually lower than 1 % in Kashi Prefecture, Possion model in Scan statistics was used to measure whether their aggregation tendency existed in space by taking the brigade as the basic unit of spatial analysis. The spatial Scan statistic can detect spatial clusters by using a variable circular window size. A total of 1999 times of Monte Carlo simulation were used to make a statistical inference of the likelihood ratio test. The risk associated to the spatial clusters is presented as the relative risk (RR), i.e. the ratio of estimated risks inside and outside the cluster. We deemed statistical differences to be significant for *P*-values lower than 0.05 .

### Quality control

In order to ensure the high access rate and the quality of interview, interviewers were trained and a preliminary investigation was performed. Before the formal investigation, a propaganda including disseminating the importance of disease hazards and prevention, and the project as well, which was led by the township government in the study areas was done in order to increase the compliance of the household in the survey. A specially-assigned person was responsible for follow-up in order to reduce withdraw bias.

## Results

### Goodness-of-fit test of the binomial distribution

A frequency distribution of the number of cases in the families is shown in Table 1. Results from goodness-of-fit test of the binomial distribution indicated that the distribution of VL cases in families did not meet the binomial distribution criteria (Table 2) as binomial distribution is usually a random distribution. Thus, preliminary determination for the distribution of VL cases with familial aggregation in this area was given out.

**Table 1:** Frequency distribution of the number of cases in the families

Number of cases	Population of families												Total
	1	2	3	4	5	6	7	8	9	10	11	13	
0	27	49	80	98	145	88	47	18	16	3	2	0	573
1	0	5	8	23	42	23	16	3	7	1	0	1	129
2		0	3	6	12	6	3	3	2	1	0	0	36
3			0	2	5	1	2	0	1	0	0	0	11
4				0	0	1	0	0	0	1	0	0	2
5					0	0	0	0	0	0	0	0	0
6						0	0	0	0	0	0	0	0
7							0	0	0	0	0	0	0
8								0	0	0	0	0	0
9									0	0	0	0	0
10										0	0	0	0
11											0	0	0
12												0	0
13													0
Number of households	27	54	91	129	204	119	68	24	26	6	2	1	751

**Table 2:** Goodness-of-fit test of binomial distribution for VL cases in families

Number of cases in households	Actual number of households	Theory number of households
0	573	542.585
1	129	177.934
2	36	27.588
3	11	2.694
≥4	2	0.189
Df	2	
χ^2^	53.230	
*P*-value	*P* < 0.01	

### Runs test

In each of the groups defined above, families with VL patients were marked as 1, otherwise, as 0. The randomness of arrangement of VL patients was analyzed by runs test (Table 3). The results showed that in the groups which had more than five families with VL patients, the distribution of patients along the ditches was not random. There were 113 families with VL patients in groups which had more than five families with patients, accounting for 63.84 % (113/177) of the total families with patients. The results showed that in queues of households arranged linearly along the ditches, the distribution of patients’ families in groups which had more than five infected families existed a partial aggregation. In groups with less than five, the aggregated distribution of families with patients was not obvious.

**Table 3:** The distribution of families with VL patients by runs test

Group number	Number of families with patient	Total households	Z-value	*P*-value
A1	6	20	−2.156	0.031
A2	10	24	−2.221	0.026
A3	10	28	−2.675	0.007
A4	18	45	−3.176	0.001
A6	16	37	−2.264	0.024
A7	9	23	−2.45	0.014
A8	9	20	−2.043	0.041
C1	17	64	−2.261	0.024
C2	10	46	−2.282	0.022
C7	8	26	−2.166	0.030

Note: This table only listed groups which had more than 5 families with a patient, where A, B and C denotes the 1th village, the 18^th^ village and 20^th^ village, respectively

### Scan statistical analysis

The distribution of families with VL patients in accordance with households’ location was analyzed by Scan statistic. The result is shown in Table 4, there were 3 gathering areas by the analysis. VL cumulative incidence was 1.95 times higher in the center of Zone A (76.153447°E, 39.528477°N) within a radius of 1.11 km and 1.82 times higher in the center of Zone B (76.111968°E, 39.531895°N) within a radius of 0.54 km, and 1.31 times in the center of Zone C (76.195427°E, 39.563835°N) than the surrounding areas, respectively.

**Table 4:** The Scan statistical analysis for VL in spatial distribution

Typical area of aggregation	The center coordinates		Radius (km)	Relative risk (RR)	Logarithm-likelihood ratio	*P*-value
	Longitude	Latitude				
A	76.153447	39.528477	1.11	1.95	5.6341	0.028
B	76.111968	39.531895	0.54	1.82	7.1678	0.005
C	76.195427	39.563835	0.68	1.31	5.7424	0.017

**Fig. 2:**
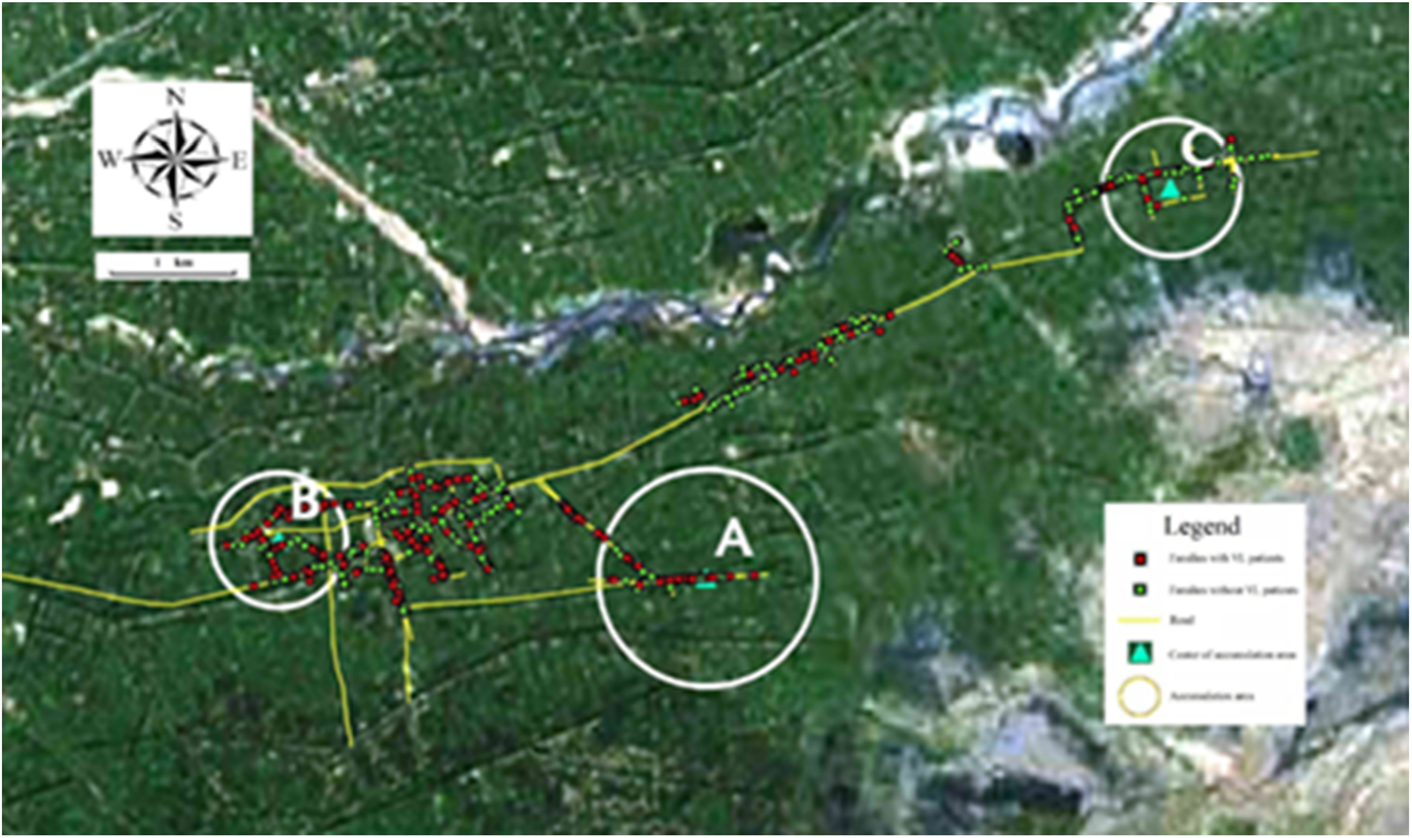
Mark of VL spatial cluster and accumulation area. Red points symbolize families with VL patients and green points symbolize families without VL patients. Yellow lines symbolize the road in villages. Zone A,B,C shows the high risk areas of VL. The bigger the calculated spatial aggregation area is, the stronger the aggregation is

The map (Fig. 2) exported by ArcGIS™ 10.1 shows the high risk areas of VL in the circle. The bigger spatial aggregation area calculated, the stronger the aggregation was.

The above-mentioned results showed that the distribution of local VL patients did not follow the spatial random distribution, but some aggregation or spatial trends from general geographical information was represented to a certain extent. This implied that there were differences among the number of VL patient in different regions. High density areas represented aggregation and trends of the disease while low density areas indicated sparseness and discreteness of the disease.

## Discussion

Leishmaniasis cases in Kashi Prefecture accounted for more than 90 % of the total number of cases in Xinjiang, especially in Bosh Klum Township of Shufu County, which accounted for 43.53 % (74/170) of the total number of cases in Kashi Prefecture and has been the most serious kala-azar endemic area in China. General investigation of 24 groups in Kashi Prefecture in 1990−2005 showed that cumulative incidence of kala-azar was 1.69–13.21 % of different geographic locations. Trend-surface analysis of this result showed there was obvious spatial clustering [16]. Out of 4,810 cumulative incidence of people surveyed, 6.44 % (310/4810) were confirmed to be kala-azar patients [17].

The distribution of parasitic infection has often been inhomogeneous [18]. Family cluster analysis can help to determine the prevalence of the diseases, determine the key targets for the control activities, establish an effective prevention and control measures with efficiency. If taking families as units in the binomial distribution analysis, and the incidence of a disease was in line with this distribution which is a random distribution, then the disease was usually considered to have no family aggregation. On the contrary, the disease would be within a family cluster. To date, this method has been used to detect the family cluster of schistosomiasis and intestinal parasites [19–21]. In this study, we used a goodness-of-fit test for binomial distribution to verify whether the VL existed familial cluster in Kashi Prefecture. The result showed that the distribution of VL in family did not meet the binomial distribution criteria, however showing a familial aggregation. The findings demonstrated that new cases seemed to occur in families which had VL patients in the past. The family cluster of VL cases may be associated with living habits of the vector, *Phlebotomus longiductus*, which had domestic and peridomestic species and were widely distributed in Kashi Oasis [22]. Research has proven that the sandflies which perched on houses and fences as their breeding habitat to complete the life-cycle [15]. This may result in family cluster of VL. Normally, after a sandfly bites a patient in the house, it inhabited in the same family and it may likely bite the other family members causing VL to spread within the same family.

One of the important features of the spatial distribution is the spatial clusters of disease and vector. The analysis of the disease spatial cluster has been one of the important epidemiological analysis methods which would be used to study the causative factor and risk factor of the disease. It helped epidemiologists to distinguish the role of random and potential element in occurrence and distribution of the disease and provide a vital basis for the cause of a disease and its influence factors [23–25]. In order to further understand the spatial cluster of kala-azar, we performed runs test and Scan statistics separately. Results were mainly embodied in two aspects: 1) in houses whose drains were linearly arranged, distribution of households with kala-azar were aggregated, and 2) in accordance with the spatial distance of household location, distribution of households with kala-azar occurred in spatial clusters. As local residential living water was snow-broth which was introduced by man-made ditches after snow melt, local residents’ houses were arranged along the ditches in line specifically. The research showed the distribution of kala-azar patients were not randomly distrubuted but accumulative in ditches which had more than five households with VL patients. In other words, families with kala-azar along ditches tend to distribute adjacently. Spatial cluster analysis showed similar conclusion in the distribution of kala-azar patients. As shown in Fig. 2, RR (Relative Risk) of kala-azar within the gathered radius was higher than outside the radius. Kala-azar patients were relatively concentrated within the radius of aggregation and sparse distribution was observed outside aggregated areas.

The distribution of kala-azar among people may have a close relationship with range of *Ph. longiductus* activities which were limited depending on whether they were domestic or peridomestic. The flight distance of sandflies was generally not more than 30 meters, but 170 meters was the farthest flight distance reported. In India, it was recorded that moving a village 100 meters away from the place where kala-azar was prevalent can prevent the transmission and spreading of kala-azar. This was described indirectly that the range of sandfly activities were limited [26]. We could not rule out the fact that infected sandflies perched on houses of kala-azar patients may fly to houses nearby and bite susceptible people because the distance between their rooms was generally within 50 meters. Thus, infected sandflies may transmit the disease to neighbors leading to a trend of accumulative distribution of kala-azar in nearby families.

In addition, there were some limitations in our design study method. We have reported that infected population of kala-azar without clinical symptoms accounted for 2.41 % (4/166) in the study area [27]. We did not take into account infected population of kala-azar without clinical symptoms in our study, but this part of the population had important significance for the potential transmission of the disease, which could influence the spatial distribution of kala-azar and may have an impact on the results of this study.

The allocation of health resources reasonably and making the limited health resources play the greatest preventive measure has been an important issue. Cluster of VL in population distribution indicated that infection was inhomogeneous in space. In our study, we found that there were some villages and townships with high incidence and others with low incidence of VL. Therefore, we can improve prevention and control of high-risk areas treat patients actively and spray indoors with insecticides to protect people in and around the families with VL patients. *Ph. longiductus* was one of the main sandflies in oasis, and widely distributed in the agricultural district of oasis. Spraying indoors with drug lagged long can effectively reduce the sandfly density as many of them are domestic [28]. This method was expected to reduce the incidence of some accumulated areas, evolve the gathering area into the sporadicones and effectively reduced the risk of the disease.

There were still some controversial opinions in the role of livestock in transmission of kala-azar, but infact it was an important influencing factor for the spatial distribution of kala-azar. However, the influence of livestock was not involved in this study. Relevant information about livestock in endemic area of kala-azar could be collected in a future study in association with human infection and distribution of patients and to explore effective control strategy of the disease.

These exsisted objective conditions show that it is still a long-term task to prevent and control Kala-azar in Kashi. Local health agencies should continue to improve the surveillance of the disease and vector control, while preventing the epidemic from relapsing.

## Conclusions

VL is a serious infectious disease and Kashi Prefecture of Xinjiang is one of the most seriously affected areas with VL in China. According to our study, the spatial distribution of VL infected families were clustered in several zones. The proper use of the findings from the study in surveillance, health resource allocation, prevention and control of VL would be an improvement in highly endemic areas. Furthermore, extending spatial analysis method of VL to other endemic areas could provide a basis and support for control and prevention of kala-azar.

## Abbreviations

CDC: center for disease control and prevention
GIS: geographical information systems
GPS: global positioning system
PKDL: post kala-azar dermal leishmaniosis
RR: relative risk
VL: visceral leishmaniasis
